# Silica Desiccant Canister: An Unusual Colonic Foreign Body

**DOI:** 10.1155/2022/9917884

**Published:** 2022-05-27

**Authors:** Rajarajeshwari Ramachandran, Vikash Kumar, Giovannie Isaac-Coss, Denzil Etienne

**Affiliations:** ^1^Department of Gastroenterology, Brooklyn Hospital Center, Brooklyn, NY, USA; ^2^Department of Internal Medicine, Brooklyn Hospital Center, Brooklyn, NY, USA

## Abstract

We are reporting a case of incidental identification and removal of two silica desiccant canisters from the cecum in a patient undergoing screening colonoscopy.

## 1. Introduction

Ingested foreign bodies rarely cause life-threatening manifestations in adults. Once the foreign body moves past the lower esophageal sphincter, which is the narrowest part of the gastrointestinal tract, it usually passes spontaneously [1]. In this case report, we present a 51-year-old man in whom two pill desiccants were identified incidentally during screening colonoscopy.

## 2. Case Description

**Figure 1:**
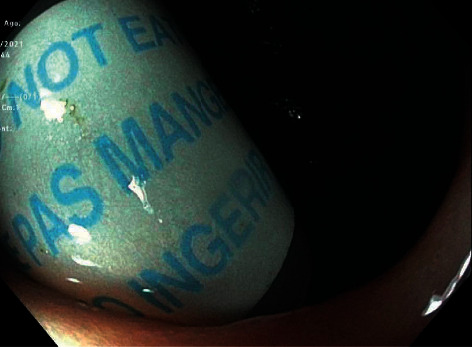
Cylindrical foreign body labeled “DO NOT EAT” in the cecum.

**Figure 2:**
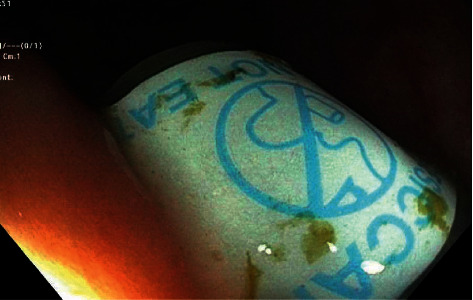
Cylindrical foreign body labeled “DO NOT EAT” in the cecum.

**Figure 3:**
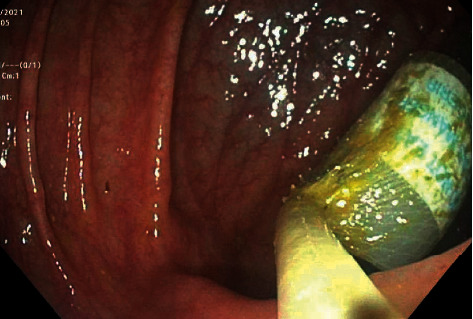
Foreign body removal using Roth Net.

**Figure 4:**
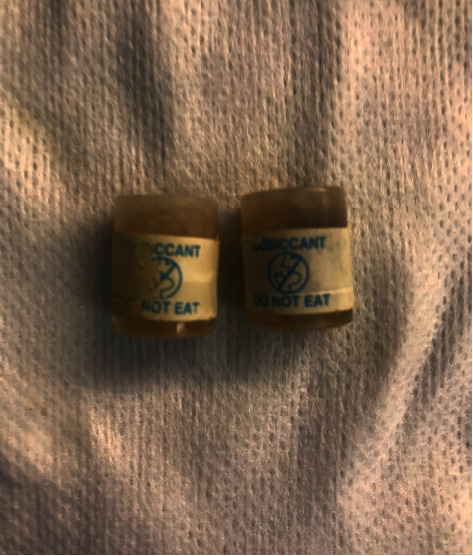
Desiccant canisters after removal.

A 51-year-old man with a past medical history of vitamin D deficiency presented to the hospital for screening colonoscopy. The patient denied nausea, vomiting, abdominal pain, altered bowel habits, or bloody stools. Colonoscopy revealed two cylindrical foreign bodies labeled as “DO NOT EAT” in the cecum (Figures 1 and 2), and the examination was otherwise unremarkable. Both foreign bodies were removed using a Roth Net (Figure 3). After removal, the foreign bodies were identified as desiccant canisters (Figure 4). In retrospect, our patient indicated that he might have ingested the desiccant canisters mistaking them for the last two vitamin D capsules out of the medication container in the days leading up to the colonoscopy.

## 3. Discussion

Ingestion of foreign bodies can be accidental or intentional. Accidental ingestion of foreign bodies is common among children and in adults with dementia and visual impairment. Foreign body ingestions are commonly asymptomatic but, in some cases, might lead to complications and, in rare cases, necessitate surgery.

Desiccants are hygroscopic substances that absorb water and are often used in medication containers to maintain dryness inside the package. Desiccants are available as packets or cylinders, and ingredients are either silica gel or calcium oxide. Silica gel is nontoxic, while calcium oxide can react with saliva, reaching a temperature of 100°C within 1 minute, and the product of this reaction is calcium hydroxide and is strongly basic (pH of 12.6) and causes chemical burns of the mucosa as reported by Hagiwara et al. [2].

Pharmaceutical companies have labeled the desiccant packaging as “DO NOT EAT.” However, as medications are manufactured in different colors and shapes and consumed by a wide demographic of patients including those with advanced age, lower educational status, language barriers, visual impairment, and memory disturbances, the “DO NOT EAT” labeling alone might not be sufficient to avoid accidental ingestion. By designing the desiccants in different colors and shapes compared to the medication, anchoring desiccants to the medication bottle, and involving the pharmacists in providing education to the patients regarding the desiccants included in the medication containers, accidental ingestion of desiccants can be reduced significantly [3].

In our literature review, we identified four patients who developed symptoms following accidental ingestion of desiccant canisters. Three elderly patients including a patient with a prior history of esophageal stricture secondary to gastroesophageal reflux disease (GERD) had a desiccant canister lodged in the esophagus causing acute hypoxic respiratory failure and were successfully treated endoscopically [3–5]. Fourth case was reported by Wu et al. in a young woman who developed abdominal pain and unintentional weight loss in the setting of chronic constipation [1]. In this patient, enteroclysis demonstrated a cylindrical foreign body proximal to the terminal ileum. Patient underwent ileocecectomy with removal of the dessicant canister and pathology specimen confirmed the diagnosis of Crohn's disease.

Our case is the first report of accidental ingestion of more than one desiccant canister, and it was identified as an unexpected colonic foreign body. We removed the desiccant canisters due to initial uncertainty regarding the nature of the foreign body and concern for patient safety given the labeling “DO NOT EAT.” However, due to the absence of anatomical narrowing in the large intestine in our patient, he would have most likely passed the desiccant canisters spontaneously in his feces.

It is likely that ingestion of desiccant canisters is a more frequent occurrence in the general population but remains largely unrecognized due to lack of symptoms and need for further investigations. The United States Census Bureau estimates patients over age 65 will outnumber children under 18 by 2034 [6]. Due to the increasing aging population and the rising prevalence of GERD which could predispose to esophageal strictures, we anticipate higher rates of symptomatic cases from accidental desiccant ingestions unless additional safety measures are taken by the manufacturers.
